# Seeing is believing: Whole-cell electron tomography models of vacuole morphology and formation in the early-stage root cortex of Arabidopsis

**DOI:** 10.1093/plcell/koaf057

**Published:** 2025-03-20

**Authors:** Yong Cui, Jiayang Gao, Yanbin Li, Hai Zhang, Xiaohui Zheng, Qing Qi, Shengqi Zhang, Byung-Ho Kang, Liwen Jiang

**Affiliations:** State Key Laboratory of Cellular Stress Biology, School of Life Sciences, Faculty of Medicine and Life Sciences, Xiamen University, Xiamen 361102, China; School of Life Sciences, State Key Laboratory of Agrobiotechnology, The Chinese University of Hong Kong, Shatin, New Territories, Hong Kong, China; State Key Laboratory of Cellular Stress Biology, School of Life Sciences, Faculty of Medicine and Life Sciences, Xiamen University, Xiamen 361102, China; State Key Laboratory of Cellular Stress Biology, School of Life Sciences, Faculty of Medicine and Life Sciences, Xiamen University, Xiamen 361102, China; State Key Laboratory of Cellular Stress Biology, School of Life Sciences, Faculty of Medicine and Life Sciences, Xiamen University, Xiamen 361102, China; State Key Laboratory of Cellular Stress Biology, School of Life Sciences, Faculty of Medicine and Life Sciences, Xiamen University, Xiamen 361102, China; State Key Laboratory of Cellular Stress Biology, School of Life Sciences, Faculty of Medicine and Life Sciences, Xiamen University, Xiamen 361102, China; School of Life Sciences, State Key Laboratory of Agrobiotechnology, The Chinese University of Hong Kong, Shatin, New Territories, Hong Kong, China; AoE Centre for Organelle Biogenesis and Function, Centre for Cell and Developmental Biology, The Chinese University of Hong Kong, Shatin, Hong Kong, China; School of Life Sciences, State Key Laboratory of Agrobiotechnology, The Chinese University of Hong Kong, Shatin, New Territories, Hong Kong, China; AoE Centre for Organelle Biogenesis and Function, Centre for Cell and Developmental Biology, The Chinese University of Hong Kong, Shatin, Hong Kong, China; Institute of Plant Molecular Biology and Agricultural Biotechnology, The Chinese University of Hong Kong, Shatin, Hong Kong, China; CUHK Shenzhen Research Institute, Shenzhen 518057, China

Dear Editor,

Vacuoles, the largest membrane-bound organelles, play crucial roles in plant growth and development, yet for more than 40 yr, significant discussion has persisted regarding their morphology and the membrane source involved in their formation (Marty 1978, 1999; Hunter et al. 2007; Viotti 2014; Shimada et al. 2018). Two distinct models of vacuole biogenesis have been proposed recently: One suggests that separate vacuoles are derived/matured from multivesicular body (MVB) fusion, while the other proposes that a single interconnected vacuole is derived from the endoplasmic reticulum (ER) (Cui et al. 2020). In Cui et al. (2019), using whole-cell electron tomography (ET) analysis of high-pressure freezing (HPF)-prepared root samples, we reported that in early developmental root cortical cells, nascent vacuoles appear as distinct small vacuoles (SVs), ranging from 400 to 1,000 nm in diameter and containing typical intraluminal vesicles (ILVs) found in the MVB, indicating that these SVs are primarily derived/matured from MVB fusion and their subsequent fusion leads to the formation of larger vacuoles. Consequently, vacuoles of different sizes function as distinct organelles within the root cortex (Cui et al. 2019). This distinct vacuole model is further supported by confocal imaging photoconversion experiments, which revealed that in living cells of both root and stomata, the photoconverted Kaede proteins were often confined to the same vacuole upon activation without dispersing to other separated vacuoles within the same cell (Cui et al. 2019; Cao et al. 2022). In addition, whole-cell ET analysis of leaf stomatal lineage cells similarly revealed the presence of distinct vacuoles, albeit big vacuoles were already detected in meristemoid (an early stomatal precursor cell), the origin of which remains unclear (Cao et al. 2022).

To address the observation of big vacuoles in early stage of stomatal cell development (Cao et al. 2022), we have now performed further whole-cell ET analysis on the root cortex initial cell and adjacent successive cells of the same root (Cui et al. 2019) to investigate vacuole morphology and formation at its earliest stages to track vacuole biogenesis along root cortex. ET results demonstrated that in the first root cortical cell (the cortex/endodermis initial cell), most vacuoles appear as distinct spherical structures with few ILVs inside, similar to those observed in meristemoid (Fig. 1, A to D, H; Supplementary Fig. S1, Supplementary Video S1). In addition, tubular vacuole structures were occasionally observed (Fig. 1, E to H). However, no connections between these tubular vacuoles and other organelles, including the ER, were observed. During cortex development, cortical cells initially undergo rapid elongation, as indicated by the gradual elongation of the second and third cortical cells (Fig. 1, I to L; Supplementary Fig. S1N). This is followed by cell division, evidenced by the sudden reduction in size of the fourth cortical cell (Fig. 1, M and N; Supplementary Fig. S1N). Meanwhile, vacuoles also first increase in size and subsequently undergo fission to accommodate these cellular changes (Fig. 1, K and L, Q; Supplementary Fig. S1N, Supplementary Videos S2 to S4). Afterward, the fourth cortical cell may undergo another cycle of elongation and division, as evidenced by the size changes of the fifth to 11th cortical cells (Supplementary Fig. S1). In the fourth cortical cell, we observed a significant number of nascent vacuoles appearing as spherical SVs containing ILVs (Fig. 1O). The absence of SVs in the cortex initial cell indicates that the SVs in fourth cortical cells are synthesized de novo and derived from MVBs, consistent with our previous study (Cui et al. 2019). Additionally, even tubular vacuoles contain ILVs, suggesting that newly formed MVBs may contribute to the extension of tubular vacuoles of young cortical cells (Fig. 1P). In addition, we also applied whole-cell ET analysis to other cell types, including epidermal cells, as well as the quiescent center (QC) and 2 successive adjacent columella cells. In the young epidermal cell, vacuoles are distinct, similar to those observed in the cortex (Fig. 2, A and B; Supplementary Video S5). In the QC and columella cells, separated spherical vacuoles were also observed (Fig. 2, C to H; Supplementary Videos S6 to S8), supporting a model of distinct vacuoles across various cell types in *Arabidopsis* root.

**Figure 1.**
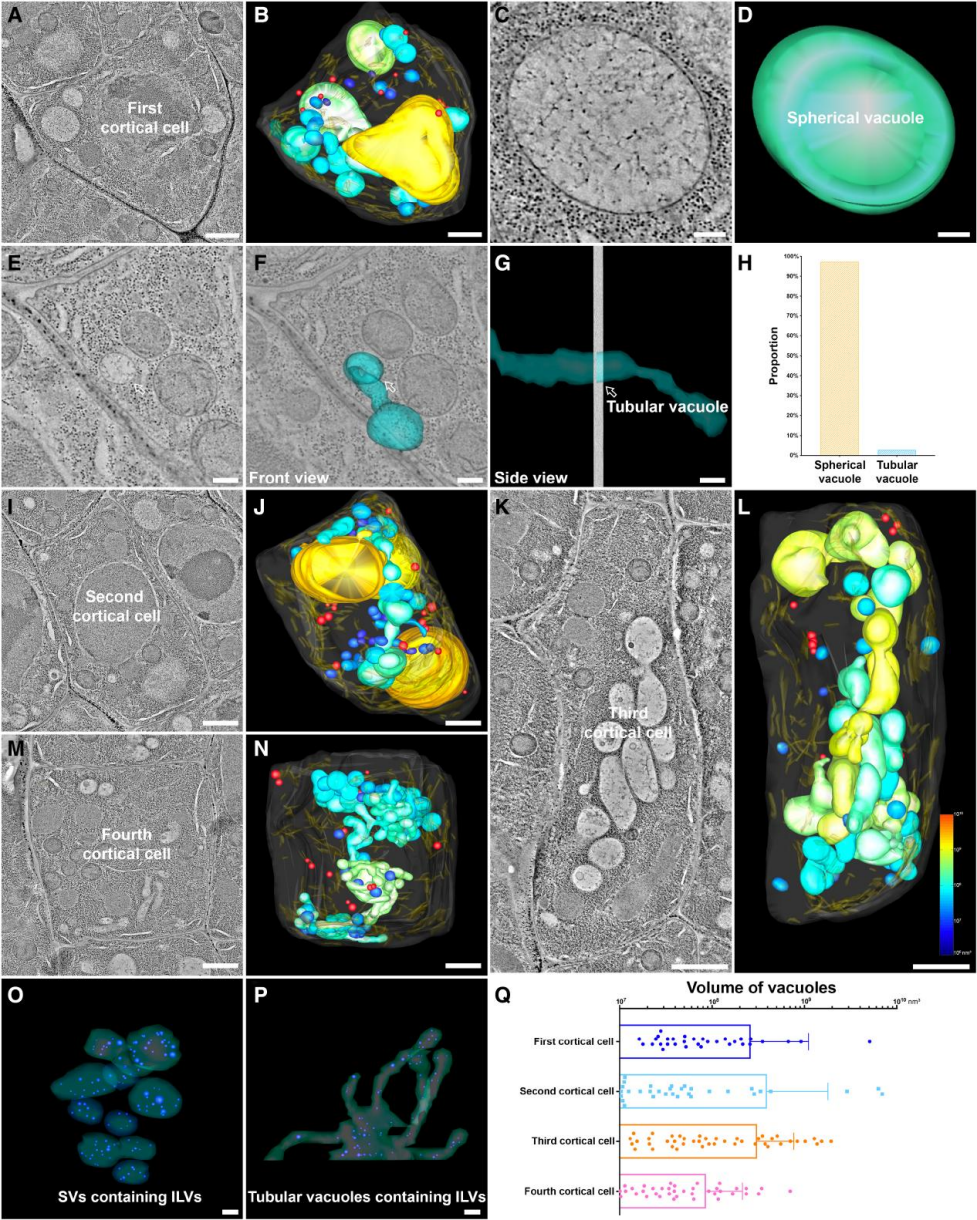
Whole-cell ET analysis of vacuoles in the early-stage root cortex. **A** to **Q)** Representative tomographic slices **(A, I, K, M)** and their corresponding models **(B, J, L, N)** illustrated the morphology of vacuoles (color-coded by volume), MVBs (red), and ER (gold) in the cortex/endodermis initial cell (first cortical cell), as well as the second, third, and fourth cortical cells. In the first cortical cell, spherical vacuoles lacked ILVs **(C** and **D)**, and the tubular vacuole showed no connections to other organelles **(E** to **G)**. In contrast, in the fourth cortical cell, numerous spherical vacuoles contained ILVs (blue), and even tubular vacuoles housed some ILVs (blue) **(O** and **P)**. Quantitative analysis revealed that most vacuoles in the first cortical cell were spherical **(H)** and the distribution of individual vacuole volumes in the first four cortical cells was assessed. Data are presented as mean with SD **(Q)**. Scale bars represent 1 *μ*m in **A** and **B)** and **I** to **N)**, and 200 nm in **C** to **G)** and **O** and **P)**. ER, endoplasmic reticulum; MVB, multivesicular body; ILVs, intraluminal vesicles.

**Figure 2.**
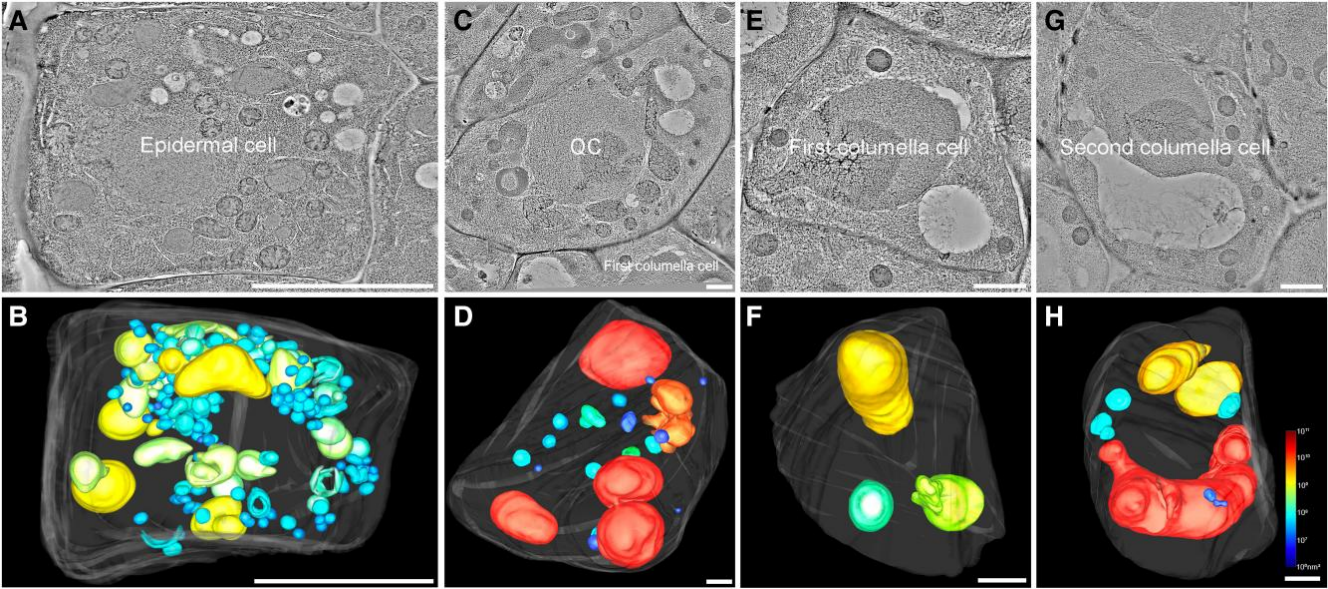
Whole-cell ET analysis of vacuole morphology in root epidermal cells, as well as in the QC and adjacent columella cells. **A** to **B)** The representative tomographic slice **(A)** and the corresponding model **(B)** illustrated vacuole morphology in a complete epidermal cell at an early developmental stage. **C** to **H)** Whole-cell ET revealed the vacuole structures in the QC **(C** and **D)** and the 2 successive adjacent columella cells **(E** to **H)**. All vacuole models are color-coded according to their volume. Scale bars represent 1 *μ*m. QC, quiescent center.

The development of an interconnected vacuole model is supported by multiple evidences in several previous studies: (i) 3D confocal imaging of interconnected vacuoles in the root meristem of both wild type and *free1* mutants (Viotti et al. 2013; Kolb et al. 2015); (ii) 2D transmission electron microscopy (TEM) identification of tubular vacuoles in root meristem (Viotti et al. 2013); and (iii) visualization of an interconnected vacuole in chemically fixed root cells, as revealed by serial block-face scanning electron microscopy (SBF-SEM) (Scheuring et al. 2016). However, our high-resolution whole-cell ET and photoconversion analyses clearly revealed distinct vacuoles in the QC, cortical, epidermal, and columella cells of the roots, as well as in the stomatal lineage cells of the leaves (Cui et al. 2019; Cao et al. 2022, and this study). In addition, previous studies have demonstrated that vacuoles are distinct organelles during the cell cycle in the shoot apical meristem of *Arabidopsis* (Seguí-Simarro and Staehelin 2006).

Recently, Scheuring et al. (2024) raised further concerns by conducting a systematic analysis of vacuole morphology at successive stages using 3D confocal imaging. Starting from the youngest cortex cells near the QC, they suggested the presence of an interconnected tubular vacuole network in the root cortex (Scheuring et al. 2024). This finding is further supported by 3D vacuole models from other cell types, including epidermal and endodermal cells. Additionally, higher-resolution STED imaging was used to demonstrate this phenomenon in a cortical cell near the QC. The vacuole-connectivity fluorescence recovery after photobleaching (vaccFRAP) method further confirmed that the thin, highly tubular vacuoles observed at the earliest stages of development form an interconnected network. Lastly, it was also argued that the presence of ILVs in the small vacuole lumen (Cui et al. 2019) may merely indicate functional endomembrane trafficking to the vacuole and could not serve as evidence supporting that MVBs are the main membrane source for vacuole biogenesis (Scheuring et al. 2024).

What could contribute to the differences of vacuole morphology observed in these studies using whole-cell ET vs confocal imaging? In addition to the previously discussed reasons (Cui et al. 2019, 2020), such as the limited resolution of conventional confocal microscopy (∼200 nm) and STED microscopy (∼50 nm), which are insufficient to resolve the distance between 2 vacuoles that can be as close as 20 nm apart as observed by 3D ET (Cui et al. 2019), several additional possibilities could be considered. For example, capturing high-quality 3D confocal images of entire cells may take several minutes, and even longer with 3D STED imaging, during which vacuoles may undergo dynamic fusion and/or fission, leading to the transient formation of connected vacuoles. Moreover, because vacuoles move rapidly in roots, the reconstructed 3D vacuole model may not accurately represent their native morphology due to the extended exposure time of several minutes. In contrast, we utilized HPF to freeze root samples within milliseconds, instantly immobilizing cellular molecules, minimizing ice crystal damage, and preserving vacuole morphology in a near-native state.

Scheuring et al. raised another concern that cutting roots during HPF could introduce mechanical stress and damage, leading to vacuole fragmentation. Interestingly, Scheuring et al. had previously employed the same HPF and even chemical fixation methods for sample preparation in their work successfully demonstrating an interconnected vacuole in *Arabidopsis* root epidermal cells (Viotti et al. 2013; Scheuring et al. 2016), but distinct vacuole model was developed using whole-cell ET analysis on HPF-prepared root samples (Cui et al. 2019). Several factors argue against the scenario of vacuole fragmentation during HPF preparation, including (i) with the latest version of HPF machine, the entire process from root cutting to freezing takes <10 s; (ii) large vacuoles remained intact and did not undergo fragmentation under ET analysis; and (iii) the young cortex region we observed is located well beyond the cut site, where vacuoles are expected to be functioning normally. Considering the similar concern, the high-intensity lasers used in FRAP and STED imaging could also induce cellular stress, potentially triggering unforeseen cellular responses, such as vacuole fusion and fission.

Scheuring and authors also questioned whether MVBs serve as the main membrane source for vacuole biogenesis. We all agree that multiple sources may contribute to vacuole formation by delivering different components to the tonoplast (Isono et al. 2010; Kruger and Schumacher 2017; Takemoto et al. 2018; Cui et al. 2020). For instance, the fusion of MVBs with vacuoles could supply phospholipid for vacuole development (Cui et al. 2014; Ebine et al. 2014; Singh et al. 2014). Another example is the direct targeting of 2 tonoplast proton pumps, H⁺-ATPase and H⁺-PPase, from the ER to vacuoles, which is crucial for the normal function of vacuoles (Viotti et al. 2013). However, compared to individual tonoplast proteins embedded in the phospholipid bilayer, the phospholipids themselves are considered the primary structural framework of the vacuole and play a crucial role in membrane organization and functionality. Among these, phosphoinositides, a group of phospholipids, are recognized as key determinants in defining membrane identity and regulating membrane dynamics (Noack and Jaillais 2017). Given that both MVBs and vacuoles share the same phosphoinositide, phosphatidylinositol 3-phosphate (Noack and Jaillais 2017), it is plausible to suggest that MVBs serve as a phospholipid source for vacuoles, positioning them as key contributors to vacuole biogenesis. Indeed, 3D vacuole models from ET in this and previous studies (Fig. 1, M and P), to Cui et al. 2019), along with 2D TEM findings, show that MVBs are abundant in early-stage root cortical cells (Fig. 3A) and participate in the de novo formation of nascent SVs. These MVBs, together with SVs, may contribute to vacuole membrane formation by directly fusing with each other (Fig. 3, B and C) or with existing vacuoles in young cortical cells (Fig. 3D), as indicated by the presence of ILVs within most vacuoles. In contrast, in the mature root cortex, large vacuoles contain few ILVs, suggesting that the increase in vacuole volume is primarily due to their homotypic fusion (Fig. 3, E and F). Thus, we conclude that vacuoles are distinct organelles in the root cortex with MVBs as the primary contributors to their biogenesis in the young root cortex.

**Figure 3.**
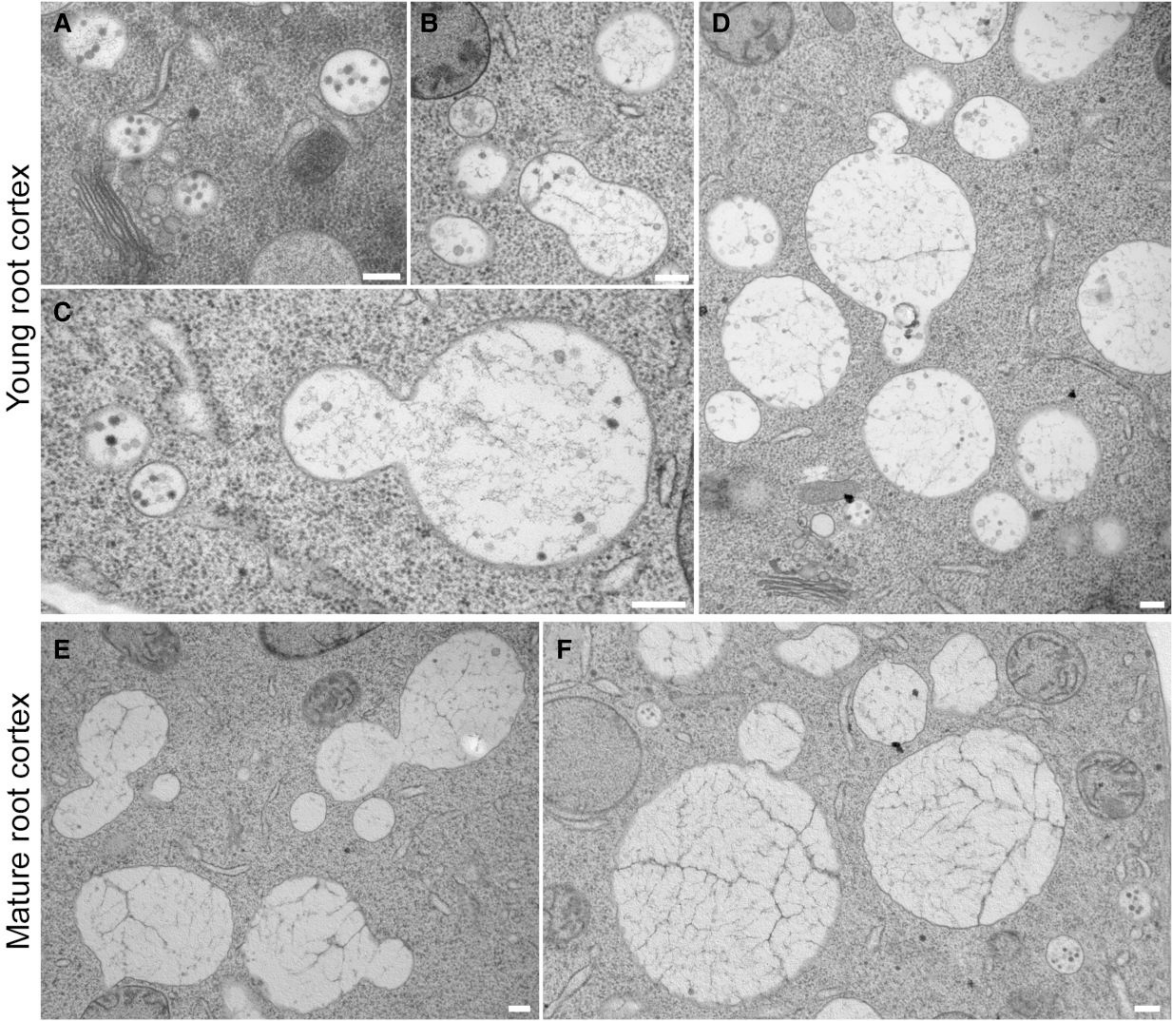
TEM analysis revealed the morphology of MVBs, SVs, and large vacuoles in both young and mature root cortex cells. **A** to **D)** A gallery of 2D TEM images displayed newly synthesized MVBs **(A)**, fusion between MVBs and SVs **(B)**, fusion between SVs **(C)**, and fusion between SVs and large vacuoles (>1 *μ*m in diameter) **(D)** in the young root cortex. **E** and **F)** In the mature root cortex, fusions between large vacuoles were frequently observed. Notably, few ILVs were present inside the large vacuoles. Scale bars represent 200 nm. MVB, multivesicular body; SV, small vacuoles.

The FRAP assay was used to demonstrate interconnected tubular vacuoles (Scheuring et al. 2024). However, one cannot exclude the possibility that this phenomenon might be attributed to frequent fusion events and rapid movement of vacuoles. In root meristematic cells, where vacuoles are small and highly dynamic, the recovery of fluorescence in bleached vacuoles may result not only from interconnections but also from fusion events between bleached vacuoles and nearby fluorescently labeled vacuoles, owing to their rapid movement. Indeed, in *vti11* (*zig1-1*) mutants, where homotypic vacuole fusion is inhibited (Surpin et al. 2003; Zheng et al. 2014), bleached vacuoles exhibit significantly slower fluorescence recovery compared to the wild type. This suggests that the rapid fusion of adjacent fluorescent vacuoles with bleached vacuoles may also contribute to their fluorescence recovery. On the other hand, since VTI11 is not involved in the formation of ER-derived interconnected vacuoles, if a single interconnected vacuole exists in the *vti11* mutant, the fluorescence recovery in this mutant would be expected to resemble that of the wild type. However, the significantly slower recovery rate of bleached vacuoles in the *vti11* mutant was observed (Scheuring et al. 2024). This phenomenon would support the MVB-derived vacuole model, as in *vti11* mutants, although homotypic fusion between mature vacuoles is inhibited, fusion between MVBs and SVs continues, resulting in the formation of separate, larger vacuoles that accumulate within the cell (Cui et al. 2019).

The interconnected vacuole model was initially proposed under the belief that the vacuole is derived from the ER, which is itself a network structure (Viotti et al. 2013). In addition to confocal imaging, Scheuring et al. (2016) demonstrated an interconnected vacuole using the SBF-SEM approach with chemically fixed samples. SBF-SEM provides significantly higher resolution compared to confocal imaging. However, a later study using the same SBF-SEM approach revealed that separate vacuoles exist in cotyledon cells of embryos (Feeney et al. 2018). In our whole-cell ET observations, tubular vacuoles are not connected to each other or other organelles, such as the ER (Fig. 1, E to G, P), leaving their origin a mystery. One possibility is that these tubular vacuoles may originate from the transformation of protein storage vacuoles (PSVs) during seed germination, as similar tubular-like vacuole structures have previously been observed in the root tip cells of tobacco seedlings (Zheng and Staehelin 2011). To further test this scenario in *Arabidopsis* roots, we conducted a time-course analysis of vacuole structures during seed germination using the TEM approach. Consistent with previous studies (Zheng and Staehelin 2011; Feeney et al. 2018), distinct spherical PSVs were observed in dry seeds (Supplementary Fig. S2A). During seed germination, PSVs tend to transform into tubular-like structures, which remain heavily stained 1 d after germination (Supplementary Fig. S2B). Between 1 and 3 d after germination, tubular-like PSVs transform into tubular-like lytic vacuoles, with their lumens shifting from heavily stained to bright (Supplementary Fig. S2, C to E). By 3 d after germination, most vacuoles, both spherical and tubular, become bright and resemble those observed at 5 d after germination (Supplementary Fig. S2F). Thus, a small number of tubular vacuoles can be observed in the root cortex, as revealed by ET (Fig. 1, E to G). Another possibility is that these tubular vacuoles may originate from the fusion of a series of SVs as demonstrated in our previous study (Cui et al. 2019). In this study, we also observed tubular vacuoles containing ILVs (Fig. 1P), further supporting this conclusion. This mechanism may also explain the phenotypes observed in the interconnected vacuole model, particularly when numerous vacuoles fuse in specific cell types and developmental stages. In addition, many other factors such as sample mounting conditions, growth media, physical manipulations, and environmental variables like light and temperature may also impact vacuole morphology. Therefore, one cannot rule out the formation of an ER-derived single vacuole under certain conditions, and thus the coexistence of multiple models of vacuole formation in plants.

In summary, “seeing is believing”, the new findings from this work not only validate the results of our previous studies but also extend the vacuole biogenesis model to the earliest stages of root cortex development, suggesting a possible vacuole fission during the early stage and followed by the de novo synthesis of SVs (Fig. 1; Supplementary Fig. S1N).

It is also important to acknowledge that every microscopy technique has its limitations and TEM is no exception. The process of cutting roots and immersing them in a nonnative medium during TEM sample preparation could introduce unpredictable cellular changes, such as alterations in vacuole shapes. In addition, the subsequent steps in TEM sample preparation—fixation, dehydration, embedding, and sectioning—could introduce artifacts, potentially affecting the native state of the specimen. While TEM provides high-resolution images, it cannot capture dynamic phenomena in real time, resulting in a lack of temporal information. For instance, the connected vacuoles observed in TEM images may represent either vacuole fusion or fission events. Therefore, findings from TEM studies are best complemented by data from optical microscopy including time-lapse movies and visualization of fluorescently tagged molecules for a more comprehensive understanding. For example, the further development and application of live-cell imaging at molecular resolution with MINFLUX instrument (Deguchi et al. 2023; Wirth et al. 2023) in plant materials would likely allow us to visualize the machinery of vacuole fusion and vacuole fission in plant cells.

We plant cell biologists are open-minded, accepting all the possibilities of scientific discoveries and come up with new hypothesis to be tested in future experiments using new technologies and systems. For example, since vacuoles have been known to undergo dramatic changes during pollen development based on 2D TEM study (Yamamoto et al. 2003), developing pollens will be an excellent singe-cell system for studying vacuole morphology, formation and function using a combination of whole-cell ET, live-cell imaging and correlative light and electron microscopy approach. Further development and application of cryo-focused ion beam (cryo-FIB) technology to thin frozen-hydrated plant specimens would be valuable, enabling cryo-ET analysis of unperturbed cells to study vacuole morphology and biogenesis in the most native state possible (Liu et al. 2021; Zeng et al. 2023). Last note, we prepare this piece soon after seeing the online commentary of Scheuring et al. 2024 without prior knowledge, but we wish that our future research efforts should be focused open-minded research and training the young scientists for future development in sciences.

## Methods

### High-pressure freezing and freeze substitution

The procedures for HPF and freeze substitution were performed as previously described (Kang 2010). Briefly, 5-d-old Arabidopsis root tips were excised and rapidly frozen using a HPF apparatus (Leica EM ICE) with 0.15 m sucrose as the filler. For ultrastructural analysis, freeze substitution was carried out in 2% OsO₄/0.5% uranyl acetate in anhydrous acetone at −80 °C for 96 h, followed by gradual warming to −20 °C over 24 h. The samples were incubated at −20 °C for 12 h and then gradually warmed to 4 °C over 4 h. After 3 rinses in acetone at room temperature, the roots were removed from the planchets and stepwise infiltrated with increasing concentrations of Epon resin (Ted Pella) over 96 h. The polymerization of the resin was performed under vacuum at 60 °C for at least 48 h.

### Electron tomography

The procedures for ET were performed as previously described (Cui et al. 2019). Briefly, 300-nm-thick sections were imaged using a Thermo Fisher Talos F200C electron microscope operated at 200 kV. For each grid, a tilt image stack (81 images) was collected from +60 to −60° with 1.5° increments. A second tilt image stack was acquired by rotating the grid by 90°. Dual-axis tomograms were reconstructed from pairs of image stacks using the etomo program from the IMOD software package (version 4.9). Fiducial markers, 15-nm gold particles on the surface of the sections, were used to align the individual images in the tilt series. After reconstruction, the IMOD software allowed for the visualization of optical slices through the 3D data in any desired orientation. The magnifications used were ×5,000 and ×14,500, corresponding to pixel sizes of 4.3 and 1.5 nm, respectively. For model generation, contours were drawn manually and meshed using the 3dmod program in the IMOD software package. To correct for section thinning during electron imaging, the *z*-scale factor was calculated to be 1.6 for the root cell tomograms. In this study, vacuoles were captured and reconstructed from a total of 15 cells, including 4 half-cortical cells, 7 partial cortical cells, 1 whole QC cell, 1 whole epidermal cell, and 2 whole columella cells.

## Supplementary Material

koaf057_Supplementary_Data

## Data Availability

The data underlying this article will be shared on reasonable request to the corresponding authors.

## Supplementary data

The following materials are available in the online version of this article.

**Supplementary Figure S1.** Overview of the QC (quiescent center) and individual root cortical cells. **A)** An illustrative diagram of the root shows various cell layers, including the cortex (green). **B** to **M)** Representative tomographic slices of the QC and root cortical cells, as indicated in panel **A**, are displayed. Scale bars represent 1 *μ*m. **N)** The working model showing vacuole morphology and de novo formation of SVs during cell elongation and division in the root cortex. Co, cortex; En, endodermis; Ep, epidermis; MVB, multivesicular body; QC, quiescent center.

**Supplementary Figure S2.** Formation of tubular-like vacuole structures in roots during seed germination. **A)** TEM images showed PSVs in dry seeds. **B** to **F)** A gallery of 2D TEM images displayed tubular-like vacuole structures at various time points after germination, as indicated. Scale bars represent 1 *μ*m. LV, lytic vacuole. PSV, protein storage vacuole.

**Supplementary Video S1.** Electron tomography analyses of vacuoles in the first cortical cell.

**Supplementary Video S2.** Electron tomography analyses of vacuoles in the second cortical cell.

**Supplementary Video S3.** Electron tomography analyses of vacuoles in the third cortical cell.

**Supplementary Video S4.** Electron tomography analyses of vacuoles in the fourth cortical cell.

**Supplementary Video S5.** Electron tomography analyses of vacuoles in the young epidermal cell.

**Supplementary Video S6.** Electron tomography analyses of vacuoles in the QC cell.

**Supplementary Video S7.** Electron tomography analyses of vacuoles in the first columella cell.

**Supplementary Video S8.** Electron tomography analyses of vacuoles in the second columella cell.
