# Supplementary material for: Seeing is believing: Whole-cell electron tomography models of vacuole morphology and formation in the early-stage root cortex of Arabidopsis
Source: Plant Cell. 2025 Mar 20;37(4):koaf057. doi: 10.1093/plcell/koaf057 (PMC11973638; doi:10.1093/plcell/koaf057)
Supplement: koaf057_Supplementary_Data [file koaf057_supplementary_data.zip › Supplementary data.pdf]

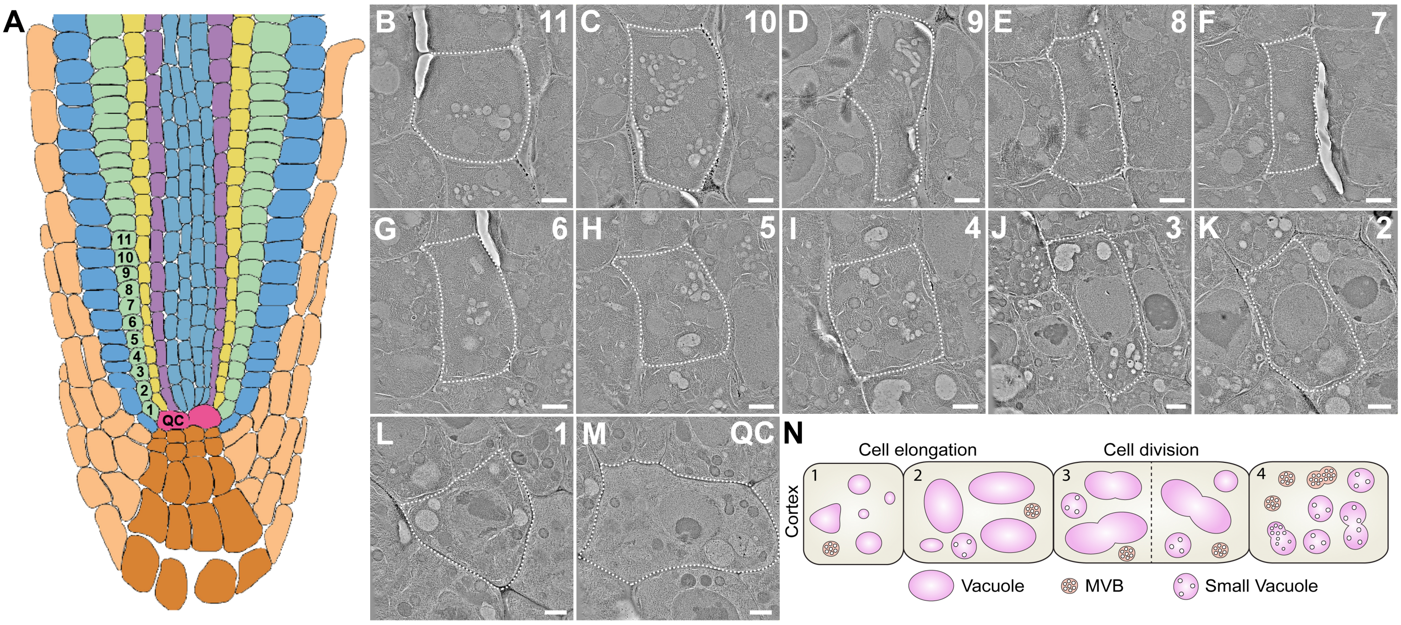

Supplemental Figure 1. Overview of the QC (quiescent center) and individual root cortical cells. A) An illustrative diagram of the root shows various cell layers, including the cortex (green). **B** to **M)** Representative tomographic slices of the QC and root cortical cells, as indicated in panel A, are displayed. Scale bars represent 1 µm. **N)** The working model showing vacuole morphology and *de novo* formation of SVs during cell elongation and division in the root cortex. Co, cortex; En, endodermis; Ep, Epidermis; MVB, multivesicular body; QC, quiescent center.

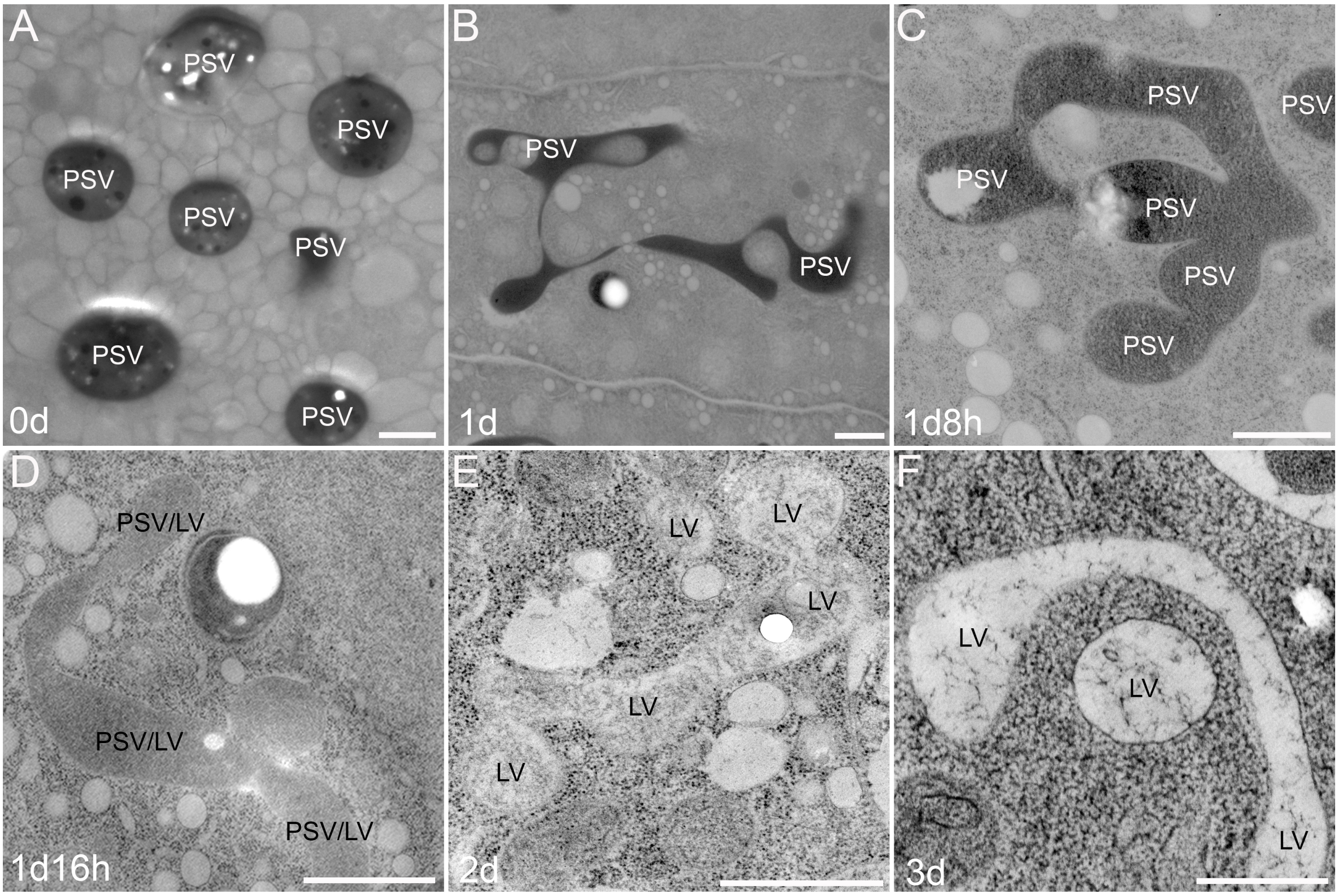

Supplemental Figure 2. Formation of tubular-like vacuole structures in roots during seed germination. **A)** TEM images showed PSVs in dry seeds. **B-F)** A gallery of 2D TEM images displayed tubular-like vacuole structures at various time points after germination, as indicated. Scale bars represent 1 µm. LV, lytic vacuole. PSV, protein storage vacuole.
